# Upregulations of Clcn3 and P-Gp Provoked by Lens Osmotic Expansion in Rat Galactosemic Cataract

**DOI:** 10.1155/2017/3472735

**Published:** 2017-11-21

**Authors:** Lixia Ji, Lixia Cheng, Zhihong Yang

**Affiliations:** ^1^Department of Pharmacology, School of Pharmacy, Qingdao University, Qingdao, China; ^2^Department of Endocrinology, People's Hospital of Weifang, Weifang, China

## Abstract

**Objective:**

Lens osmotic expansion, provoked by overactivated aldose reductase (AR), is the most essential event of sugar cataract. Chloride channel 3 (Clcn3) is a volume-sensitive channel, mainly participating in the regulation of cell fundamental volume, and P-glycoprotein (P-gp) acts as its modulator. We aim to study whether P-gp and Clcn3 are involved in lens osmotic expansion of galactosemic cataract.

**Methods and Results:**

In vitro, lens epithelial cells (LECs) were primarily cultured in gradient galactose medium (10–60 mM), more and more vacuoles appeared in LEC cytoplasm, and mRNA and protein levels of AR, P-gp, and Clcn3 were synchronously upregulated along with the increase of galactose concentration. In vivo, we focused on the early stage of rat galactosemic cataract, amount of vacuoles arose from equatorial area and scattered to the whole anterior capsule of lenses from the 3rd day to the 9th day, and mRNA and protein levels of P-gp and Clcn3 reached the peak around the 9th or 12th day.

**Conclusion:**

*G*alactosemia caused the osmotic stress in lenses; it also markedly leads to the upregulations of AR, P-gp, and Clcn3 in LECs, together resulting in obvious osmotic expansion in vitro and in vivo.

## 1. Introduction

Sugar cataract is the major ocular complication of diabetes, which seriously impairs the ocular function of diabetic subjects [1]. Hyperglycemia is thought to be the underlying factor of diabetic cataractogenesis [2]. Lens osmotic expansion, a crucial and reversible process, greatly triggers the rapid onset and accelerates the maturation of true cataract [3]; thus, this is the critical stage for the prevention of diabetic cataract. Cell swelling is the main feature of lens osmotic expansion; several proteins such as AR, P-gp, and Clcn3 are proposed to participate in this pathological mechanism.

AR catalyzes the NADPH-dependent reductions of a broad variety of aldehydes or ketones to their corresponding alcohols [4–6]. AR is the rate-limiting enzyme of polyol pathway; its overactivation induced by hyperglycemia is the blasting fuse of diabetic cataract [6]. Both galactose and glucose belong to AR substrates, but AR has higher affinity to galactose than to glucose, and galactitol is even more difficult to be further metabolized than sorbitol, so galactosemic cataract formed more rapidly than diabetic cataract model [3, 7, 8]. Although galactosemic cataract and diabetic cataract are together called “sugar cataract” [7], most researchers prefer to use galactose-induced cataract model to study the pathological mechanism of lens osmotic expansion [1, 9–11].

In lenses, AR is primarily located in LECs, and its biologic activity in LECs is approximately 21-fold of that in cortical fibers [12]. In physiological conditions, almost no glucose enters polyol pathway; but it will be markedly activated by hyperglycemia and even more than 30% glucose enters polyol pathway when blood glucose level is over 11.1 mmol/l [4, 13]. Activated AR converts amount of glucose to sorbitol, and the later does not readily diffuse across cell membrane and is poorly further reduced by sorbitol dehydrogenase [4, 13]. Therefore, more and more hypertonic sorbitol accumulates in cytoplasm and absorbs water, leading to gradual swelling of lenticular cells [13]. In lenses, perhaps AR activation originally acts as homeostatic regulation against the high osmolality of extracellular glucose; nothing but the overactivation of AR causes the accumulation of a large majority of sorbitol accompanied by influx of much water, so LECs gradually swell and eventually cause cell rupture.

Modulation of cell volume is highly conservative in evolution to maintain constant cellular volume by regulating internal ingredients to cope with the changed osmolarity [14, 15]. Despite a matter of debate existed, most investigators thought that modulation of cell volume mainly depended on the coordination of P-gp and Clcn3 [15, 16].

P-gp, a highly conserved membrane protein, is a member of ATP-binding cassette (ABC) transporter protein super family [17–19]. In humans, P-gp is encoded by the multidrug resistance gene 1 (MDR 1); in rodents, mdr1a and mdr1b genes are homologous to human MDR1 [17–19]. P-gp has already been approved to have bimodal functions; it not only serves as the transmembrane transporter being responsible for the efflux of multidrugs in isotonic condition but also switches to a modulator of Clcn3 in hyperosmotic environment [20, 21]. Reversible phosphorylation is the possible mechanism for functional shuttle of P-gp [16]. Upregulation of P-gp is closely linked to overactivated Clcn3 [10, 16], which is a volume-regulated chloride channel. Clcn3 is highly associated with the regulation of fundamental volume in response to cell swelling or shrinking [15]. The opening of Clcn3 allows the efflux of diverse osmolytes such as several amino acids and polyols, leading to concomitant decrease of cell volume [16, 22]. Anti-P-gp monoclonal antibody could directly combine distinct epitopes of P-gp and obviously inhibited Cl^−^ conductance induced by cell swelling [23]. P-gp also expressed in lens [24] and was markedly upregulated in LEC layer isolated from 30% galactose-fed rats on the 4th and 7th days [10]. Besides, P-gp transgenic hamsters could develop cataracts resembling those in mice with congenital osmotic cataract [25]. P-gp should be considered as a modulator in lens osmotic expansion of sugar cataract [24]. However, administration of AR inhibitors to galactose-fed rats could reverse the upregulation of P-gp, indicating a potential pathophysiological linkage between the osmotic stress and P-gp stimulation [10].

On the basis of the previous theories and results, we hypothesize that lens osmotic expansion, imposed by AR overactivation, would stimulate Clcn3 and its modulator P-gp to delay the lenticular swelling.

## 2. Materials and Methods

### 2.1. Primary Culture of Beagle Dog LECs

The whole eyeballs were immediately enucleated from beagle dogs after being sacrificed; we soaked them into 75% ethanol for 10 minutes, carefully isolated the lens by posterior method, and cut the lens capsule into two parts at the equatorial zone. Due to the specific distribution of monolayer LECs below the anterior capsule of lens, all anterior capsules were collected, cut into tiny fractions, moved into a flask, and gently shook to spread evenly on the bottom. In order to increase the adherence, the flask was firstly cultivated in 100% bovine fetal serum (FBS; Gibco, Auckland, New Zealand) in an erect position for 4 hours, then followed by being positioned horizontally. 24 hours later, we added 5 ml Dulbecco's modified Eagle medium (DMEM; Hyclone, Beijing, China) supplemented with 15% FBS, 2 mM glutamine, 100 *μ*g/ml streptomycin, and 100 U/ml penicillin. Another 24 hours later, we observed that many LECs were growing below or around the capsule tissue fractions, the 3rd to 8th passages of LECs were used in following studies.

### 2.2. Osmotic Expansion of LECs Induced by Gradient Galactose

The LECs were harvested and seeded on 6-well plates (20,000/well); 24 hrs later, galactose was added to final concentrations of 0, 10, 20, 30, 40, 50, and 60 mM. After 48-hour cultivation, we observed LECs' morphology under inverted microscope and harvested them. One part was used to extract total mRNA and detect mRNA expression by real-time RT-PCR; another part was used to extract total protein and measure targeted genes' protein level by Western blot. For the assessment of cell viability, LEC cultivation was carried out on 96-well plates, and cell viability was monitored by using CCK-8 reagent (Dojindo, Japan).

### 2.3. Induction of Rat Galactosemic Cataract and Assessment of Lens Opacification [26]

Male Sprague-Dawley rats (21 days old) with average body weight of 40 ± 4.07 g were purchased from the laboratory animal center at Qingdao Food and Drug Administration (SCXK-Lu-20140001; Qingdao, China). They were treated according to the ARVO statement for the Use of Animals in Ophthalmic and Vision Research. All the rats were randomized into two groups (*n* = 24), and they had free access to AIN-93 stock diet. While animals drank different water, rats in normal group drank pure water, but rats in control group drank 12.5% galactose solution in the first 7 days and 10% galactose solution from the 8th day to the end; the whole duration was 18 days. Every 3 days, lens opacification was evaluated (without anesthesia) by using a hand-held slit lamp (SL-15; Cowa, Japan) proceeded after full mydriasis with tropicamide hydrochloride. Cataract severity was scored by the same experienced observer who was blinded to the identity of each individual animal. Lens opacification was graded into five stages as follows [8, 26]: grade 0, clear normal lenses; grade 1, vacuoles, located in the cortex, cover less than one-third of the lens anterior segment, forming a subcapsular cataract; grade 2, vacuoles cover approximately two-thirds of the lens anterior segment; grade 3, diffuse opacity in cortex with/without some vacuoles; grade 4, diffuse opacity in cortex and moderate nuclear opacity; grade 5, mature milky cataract is observed as a dense opacity in both cortex and nucleus. After assessment of sugar cataract, four rats of each group were sacrificed with CO_2_ on the day of 3, 6, 9, 12, 15, and 18, respectively. Each time, we promptly isolated the intact lenses by posterior approach, weighed them, and observed the lens shape and transparency in vitro. At last, all the lens samples were thrown into lipid nitrogen and stored at −80°C until further assay.

### 2.4. mRNA Expressions of AR, P-Gp, and Clcn3 in LECs and Lenses

Total RNA was extracted from LECs or lenses with TRIzol reagent (TaKaRa, Dalian, China) on the basis of manufacturer's protocol. To avoid amplification of genomic DNA sequences, all RNA samples were treated with DNase I and diluted to 100 ng/*μ*l. 2.5 *μ*g total RNA of each sample was reverse-transcribed into first strand cDNA with the iScript cDNA Synthesis Kit (Invitrogen, Waltham, MA, America) as described by the manufacturer. mRNA quantification was performed by using SYBR Green PCR Kit (TaKaRa, Dalian, China); all reactions were carried out in a single tube reaction setup on the ARI7000 real-time PCR system. The temperature profile was as follows: stage 1, 10 s at 95°C for denaturation of cDNA/RNA hybrid; stage 2, 40 cycles of 5 s at 95°C and 34 s at 61°C; and stage 3, 15 s at 95°C, 1 min at 60°C and 15 s at 95°C. *β*-Actin, a housekeeping gene, was chosen as an internal control to normalize the expressions of target genes. A control PCR reaction involving only Taq polymerase and the primer combination was used as a negative PCR control. Specific primers were designed based on published sequences (GenBank), *β*-actin sense, 5′-ACTCTTCCAGCCTTCCTTC-3′, and antisense, 5′ATCTCCTTCTGCATCCTGTC-3′; AR sense, 5′-GTG ACC GAG GCT GTG AA-3′, and antisense, 5′-AGA GGG TTG AAG TTG GAG A-3′; P-gp sense, 5′-GCC CAT CCT GTT TGA CTG-3′, and antisense, 5′-CGC TTC CTG GAC GAC CTT-3′; and Clcn3 sense, 5′-ACA CTG ACG GGA TTG G-3′, and antisense, 5′-AGG CAT ACG GAG CAA-3′. Results were expressed as fold change of target genes versus *β*-actin by ΔΔCt method.

### 2.5. Protein Detection of AR, P-Gp, and Clcn3 by Western Blot

LECs or lens capsules were homogenized in ice-cold lysate buffer containing 2 mM EDTA, 50 mM Tris-phosphate, 150 mM NaCl, 1% NP-40, 0.5% sodium deoxycholate, 0.1% SDS, 1 mM PMSF, and 1% protease inhibitor cocktail (Nakarai Tesque, Kyoto, Japan). Tissue samples were firstly subjected for sonication (40 W, 30 minutes) on ice and then subsequently harvested the supernatants after centrifugation at 20,000*g* for 40 minutes at 4°C. Protein level was determined by bicinchoninic acid (BCA) method. Proteins (100 mg per sample) were run on a 10% gradient sodium dodecylsulfate-polyacrylamide electrophoresis gel, then transferred on polyvinylidene difluoride (PVDF) membrane (Bio-Rad, Hercules, CA, America). After blocking by TBS-T (0.01 mol/l TBS with 0.1% Tween 20) containing 5% (*w*/*v*) nonfat milk, the blots were incubated with specific primary antibodies at 4°C, such as 12 hrs with anti-goat polyclonal AR antibody (sc17736, dilution 1 : 1000; Santa Cruz, CA, America), or 48 hrs with anti-mouse monoclonal P-gp antibody (sc59593, dilution 1 : 200; Santa Cruz), or 12 hrs with anti-mouse monoclonal Clcn3 antibody (sc390010, dilution 1 : 1000; Santa Cruz). After washing by TBS-T, the membranes were incubated with anti-goat/mouse/rabbit IgG-labeled with horseradish peroxidase (dilution 1 : 2000; Santa Cruz) for 60 min at room temperature. These membranes were washed again with TBS-T and visualized by the enhanced chemiluminescence method using LAS-3000 imaging system (Fuji Photo Film, Tokyo, Japan). Exposure times ranged from 20 to 120 sec based on different proteins.

### 2.6. Statistical Analysis

All the data were presented as mean ± standard deviation (SD); the grade of sugar cataract was analyzed by Mann–Whitney test, and other data were evaluated with one-way ANOVA (two-tailed test). *p* value less than 0.05 was considered to be statistically significant.

## 3. Results

### 3.1. Effects of Galactose on LECs' Morphology

**Figure 1:**
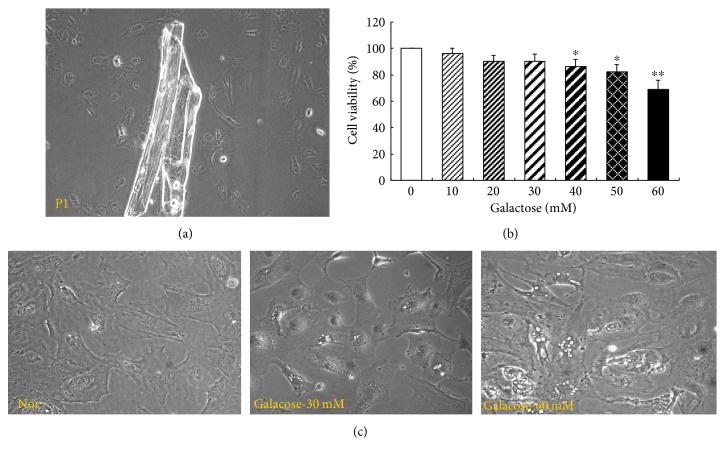
Primary culture of LECs and osmotic expression induced by gradient galactose. (a) Primary culture of beagle dog LECs (×100), P1 means the 1st passage of LECs; (b) effects of galactose on LEC viability, galactose concentration is 10, 20, 30, 40, 50, and 60 mM; (c) morphological characteristics of LECs induced by gradient galactose (×200). ^∗^ *p* < 0.05, ^∗∗^ *p* < 0.01 versus normal group.

In primary culture, fractions of lens anterior capsule were tightly attached to the flask bottom in 24 hours; some LECs were growing below or around lens capsule fractions after 48-hour cultivation (Figure 1(a)). Most oval LECs had typical epithelial-like structure with big nuclear and abundant cytoplasm. In the following passages, several LECs would switch to shuttle-like fiber cells, so we only used the 3rd to 8th passages of LECs in further studies. As to the osmotic expansion of LECs (Figure 1(c)), lots of tiny vacuoles appeared around the nucleus in the LEC cytoplasm under the coincubation with galactose in 48 hours. The higher the galactose concentration was the more the tiny vacuoles appeared. This phenomenon never appeared in normal LECs in 48-hour incubation. Our results were consistent with similar observations reported in dog LECs cultured in medium containing 3-fluoro-3-deoxy-galactoseactose [27]. As to cell viability (Figure 1(b)), only high-level galactose (>40 mM) could significantly inhibit the cell viability compared with the normal level.

### 3.2. mRNA Expressions and Protein Levels of AR, P-g, and Clcn3 in LECs Induced by Galactose

**Figure 2:**
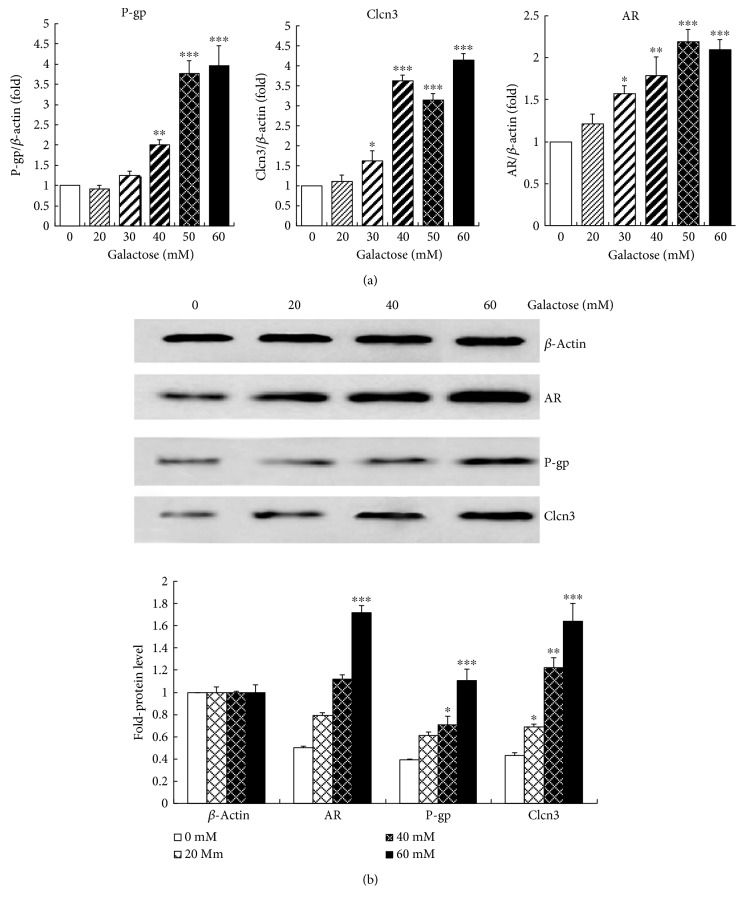
mRNA expressions and protein levels of AR, P-gp, and Clcn3 in LECs' osmotic expansion induced by gradient galactose. (a) mRNA expressions of target genes including AR, P-gp, and Clcn3; (b) Western blot and analysis of AR, P-gp, and Clcn3. ^∗^ *p* < 0.05, ^∗∗^ *p* < 0.01, and ^∗∗∗^ *p* < 0.001 versus the normal level of the same day.

As shown in Figure 2(a), CT value of AR mRNA expression was about 18.5 in normal group cultivated in DMEM containing 1000 mg/l glucose, but the expressions of P-gp and Clcn3 mRNA were almost undetected. In comparison with the normal level, AR initially increased to 1.51-fold at 30 mM galactose (*p* < 0.05), then gradually increased to 2.24-fold at 50 mM (*p* < 0.001) and kept the peak at 60 mM. 30 mM galactose markedly increased the mRNA level of Clcn3 to 1.62-fold (*p* < 0.05) and it arrived at 4.15-fold peak at 60 mM (*p* < 0.001). As to P-gp, the modulator of Clcn3, its mRNA level obviously increased to 2.01-fold at 40 mM (*p* < 0.05) and reached the peak of 3.89-fold at 60 mM (*p* < 0.001). On cellular level, AR, P-gp, and Clcn3 could be activated by high level galactose and their responses to galactose were almost consistent.

Protein levels of AR, P-gp, and Clcn3 were summarized in Figure 2(b). Although LECs were incubated in gradient galactose levels, *β*-actin expressed very stably. Proteins of AR, P-gp, and Clcn3 expressed weakly in normal medium with glucose (1000 mg/l), but they were followed by consistent rises as the galactose concentration gradually increased from 0 to 60 mM. In normal group, protein levels of AR, P-gp, and Clcn3 were only 0.50-fold, 0.39-fold, and 0.43-fold of *β*-actin, respectively, and they gradually increased to 1.71-fold, 1.10-fold, and 1.64-fold of *β*-actin in 60 mM galactose incubation, respectively.

### 3.3. Onset and Formation of Galactosemic Cataract in Rats

**Figure 3:**
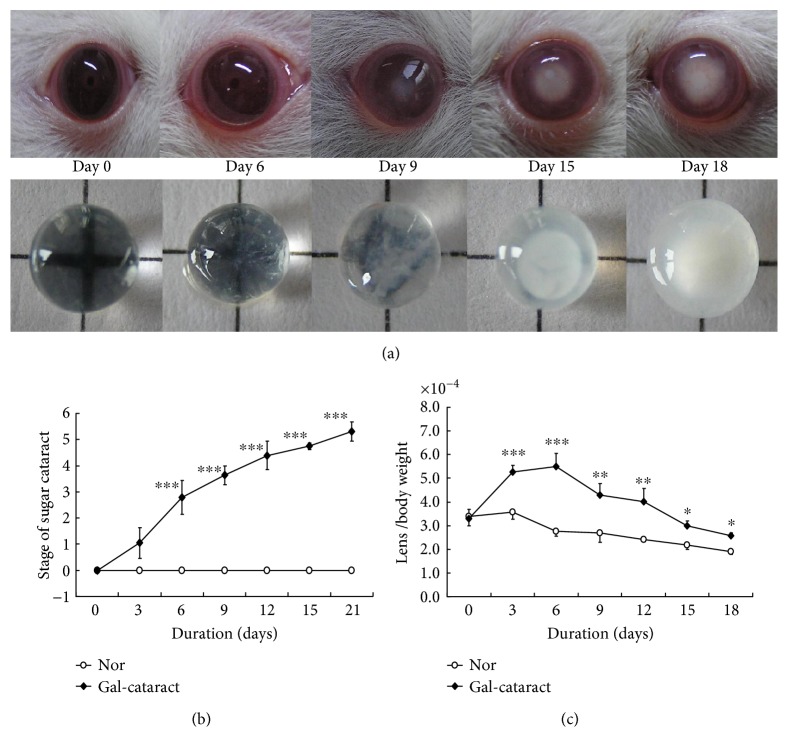
Onset and formation of rat sugar cataract induced by galactose. (a) Lens opacification of galactosemic cataract on the day of 0, 6, 9, 15, and 18; (b) grade of rat galactosemic cataract; and (c) the ratio of lens weight to body weight. ^∗^ *p* < 0.05, ^∗∗^ *p* < 0.01, and ^∗∗∗^ *p* < 0.001 versus normal level.

As shown in Figures 3(a) and 3(b), normal lenses had always been at grade 0 and isolated lenses were transparent in the whole duration, but lens opacification of galactose group gradually increased by almost one grade every 3 days. In early six days, more and more tiny vacuoles continually appeared in the periphery of lens, subsequently extended to the whole anterior capsule with or without expanded “Y” in the central area. By the 6th day, tiny vacuoles occupied over two-thirds of the anterior capsule; the average cataract grade was 2.27, and we observed that isolated galactosemic lenses began to be blurry. Although we decreased galactose concentration from 12.5% to 10% from the 8th day, lens opacification was persistently aggravated in the following days. At the 9th day, slight nuclear opacification appeared in about 80% lenses of the control group. And at the end of the 18th day, more than 85% lenses of control group were milky both in the lens cortex and in the nuclear; most of galactosemic lenses were at stage 5.

As to the ratio of lens weight to body weight (Figure 3(c)), the ratio of normal 21-day SD rat was about 3.37 × 10^−4^; it slowly decreased as normal rats grew up, but the ratio of cataractous rats obviously increased in the first 6 days and gradually decreased in the following 9 days. The maximal ratio of galactosemic rats was 5.51 × 10^−4^ on the 6th day, but the normal level was only 2.76 × 10^−4^; the control ratio was still higher (2.76 × 10^−4^) than the normal level (1.98 × 10^−4^) at the end of day 18.

### 3.4. mRNA Expressions and Protein Levels of AR, P-Gp, and Clcn3 in Rat Lenses

**Figure 4:**
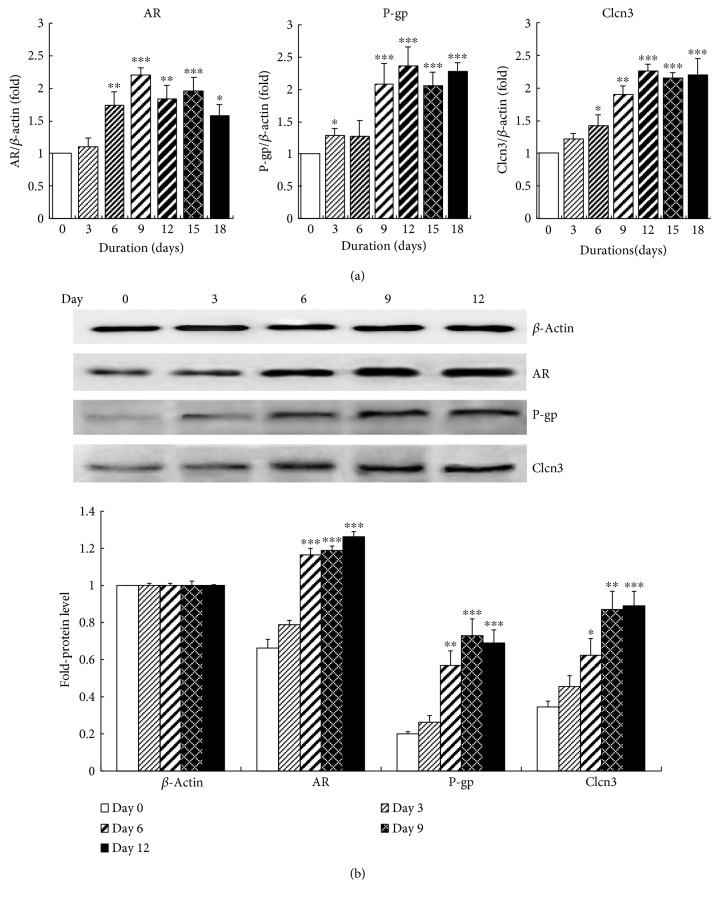
mRNA expressions and protein levels of AR, P-gp, and Clcn3 in lenses of galactosemic cataract rats. (a) mRNA expressions of target genes including AR, P-gp, and Clcn3 and (b) Western blot and analysis of AR, P-gp, and Clcn3. ^∗^ *p* < 0.05, ^∗∗^ *p* < 0.01, and ^∗∗∗^ *p* < 0.001 versus the normal level of same day.

mRNA expressions of AR, P-gp, and Clcn3 were summarized in Figure 4(a). AR, a key enzyme of polyol pathway, was gradually upregulated from the 3rd day (1.11-fold) to the peak (2.21-fold) at the 9th day, and then, it slowly reduced to 1.57-fold at the end of 18 days. These results almost coincided with our previous study [26]. The mRNA changes of P-gp and Clcn3 were similar, both P-gp and Clcn3 were gradually upregulated from the beginning to the highest level of 2.36-fold and 2.25-fold at the 12th day, respectively; then, they maintained their high levels to the end.

Since elevation of gene transcription was not always coincident with gene translation, we further examined the protein levels of target genes including AR, P-gp, and Clcn3 by Western blot (Figure 4(b)). Our analysis revealed that protein level of *β*-actin had been always on the similar level for the whole duration. On day 0, AR protein was about 0.66-fold of *β*-actin, but only faint protein expressions of P-gp and Clcn3 were detected in rat lenses; they were 0.20-fold and 0.34-fold of *β*-actin, respectively. P-gp and Clcn3 could be significantly upregulated along with the accumulation of galactitol. Protein level of AR obviously increased to 1.18-fold of *β*-actin by the 9th day, and it kept on rising to the peak of 1.26-fold of *β*-actin on the 12th day. Clcn3 and P-gp also continuously increased to the highest levels of 0.89-fold (day 12) and 0.73-fold (day 9), respectively. Based on the above results, the increased protein expressions of AR, P-gp, and Clcn3 were associated with their elevated mRNA levels.

## 4. Discussion

Diabetes is predicted to be about 552 million up to 2030 [28]; the majority of diabetic subjects are susceptible to develop sugar cataract over 10 years later. Differing from aging cataract, lens osmotic expansion occurring in early stage is the key pathological mechanism of diabetic cataract [2]. Furthermore, lens osmotic expansion also induces serious oxidative stress, accelerating the maturation of sugar cataract. In fact, overactivation of AR in lenses resulted from hyperglycemia is the original cause, which leads to the accumulation of sorbitol and severe imbalances of electrolyte and water and further stimulates homeostasis system to reverse the swelling volume of lenticular cells. The related proteins involved in osmotic expansion are AR, Clcn3, and its modulator P-gp.

On cellular level, high level of galactose (≥40 mM) could induce more tiny vacuoles appearing in cytoplasm of LECs with decreased cell viability. The severity of vacuoles was tightly associated with galactose concentration. Similar phenomenon also existed in the lenses of rat galactosemic cataract model in vivo, and lots of vacuoles initially appeared around the lens equator and continuously expanded to anterior capsule during the early stage of sugar cataract. These were the factual manifests of lens osmotic expansion induced by galactose in vitro and in vivo; the following observations of mRNA and protein expressions demonstrated the potential mechanism.

But why did vacuoles firstly appear in LECs around the lens equator in rat galactose-induced cataract model? Perhaps it is related to the distribution of LECs. LECs are a monolayer of cubical epithelial cells located below the anterior capsule of the lens; they can consistently differentiate into fiber cells at the equatorial area. LECs in the equator are more active with stronger viability of proliferation and differentiation than other LECs. Therefore, they are more sensitive to hyperglycemia to form amount of dense vacuole clusters, initiating the development of sugar cataract. Above of them are just our speculation, the precise mechanism requires further investigations.

As to rat galactosemic cataract model, few tiny vacuoles arose around the equatorial area from the 3rd day; subsequently, dense clusters of vacuoles began to expand to the anterior capsule by day 6. Meanwhile, the ratio of lens weight to body weight increased dramatically to a crazy level, which indicated that an obvious osmotic expansion came into being in galactosemic lenses. RT-PCR results also presented the significant upregulations of AR, P-gp, and Clcn3 mRNA levels at the 6th day. Along with the ongoing of galactosemic cataract, most galactosemic rats began to enter the stage of nuclear opacification from the 12th day, so the early stage was the key period to investigate the dysregulations of AR, P-gp, and Clcn3 associated with lens osmotic expansion.

Maintenance of constant volume is the physiological function of Clcn3 in many types of mammalian cells [15, 16]. The followed cell swelling results from sorbitol accumulation in LECs; lenticular cells try to restore their original volume by activating chloride conductance [15]. P-gp, as the regulator of Clcn3, participates in the regulation of Clcn3 activity [16]. The present study showed that the dysregulations of Clcn3 and P-gp were almost synergetic in response to the lenticular swelling in LEC osmotic expansion and onset of rat galactosemic cataract. Although Clcn3 and P-gp expressed faintly in normal lens anterior capsules under physiological condition, both were predominantly upregulated in galactosemia, and the early stage was a key period of lens osmotic expansion.

In conclusion, AR was overactivated by galactosemia and led to severe osmotic expansion in lenticular cells; subsequently, P-gp and Clcn3 were both upregulated due to lenticular cell swelling and tried to maintain the osmotic balance within galactosemic lenses. AR, P-gp, and Clcn3 participated in the onset and formation of lens osmotic expansion in early stage of sugar cataract. We expect that dysregulations of the above genes can provide some information for rational development of drugs against galactosemic or diabetic cataract.
